# Moracin D Inhibits Gastric Cancer Progression Through B-Cell Lymphoma-2 (Bcl-2)-Mediated Cell Cycle Arrest and Apoptosis, Enhancing Chemotherapy Efficacy

**DOI:** 10.3390/biom16030428

**Published:** 2026-03-13

**Authors:** Abdulkareem Qasem Moqbel, He Yang, Shunhui Liu, Li Feng, Muhammad Usman Ghani, Xiaoxue Ke, Hongjuan Cui

**Affiliations:** 1 Medical Research Institute, State Key Laboratory of Resource Insects, Southwest University, Chongqing 400715, China; kareem.moqbel@taiz.edu.ye (A.Q.M.); gjxy2023005@email.swu.edu.cn (H.Y.); fengli1235@email.swu.edu.cn (L.F.); kexx@swu.edu.cn (X.K.); 2 Jinfeng Laboratory, Chongqing 401329, China; muhammadusmanghani@jflab.ac.cn

**Keywords:** moracin D, gastric cancer, Bcl-2, apoptosis, targeted therapy, combination therapy

## Abstract

Gastric cancer (GC) is a highly prevalent and rapidly progressing cancer with a poor prognosis, primarily due to chemoresistance and treatment-related toxicity. Moracin D (MD), a benzofuran extracted from *Morus alba* L., has shown potential antitumor effects in various malignancies, although its impact on GC remains limited. The aim of this study was to assess the anticancer potential of MD in human gastric cancer cell lines and subcutaneous xenograft models. We examined cell proliferation, clonogenic ability, cell cycle progression, and apoptosis using MTT, BrdU, colony formation assays, flow cytometry, Western blotting, and immunohistochemistry. Our findings suggest that MD selectively inhibited GC cell proliferation and reduced DNA synthesis in vitro. It also inhibited colony formation and tumor growth in vivo, affecting GC cell clonogenicity without affecting body weight or vital organs, and without overt toxicity under the experimental conditions tested. Mechanistically, MD was found to induce G_2_/M cell-cycle arrest, potentially through modulation of cyclin B1 and CDK1, and to trigger apoptosis in GC cells, which may involve the mitochondrial pathway as suggested by changes in Bcl-2 and pro-apoptotic protein levels. While Bcl-2 overexpression partially reversed MD-induced inhibition of proliferation and apoptosis, further studies are required to confirm its role as a mediator. Additionally, MD enhances the anticancer effects of 5-fluorouracil (5-FU) through synergistic mechanism. This study highlights the observed antiproliferative and proapoptotic effects of MD in preclinical models and suggests its potential as monotherapy or in combination with 5-FU as a promising therapeutic approach in the treatment of gastric cancer.

## 1. Introduction

Gastric cancer (GC) is the most common malignancy worldwide, ranking fourth in cancer-related mortality [1]. Treatment options for GC include surgical resection, chemotherapy, radiation, immunotherapy, and precision-targeted therapies. Despite these options, the 5-year survival rate for patients with advanced GC remains below 40% [1]. Treatment is based on the standard cytotoxic agents [(oxaliplatin and 5-fluorouracil (5-FU)], but their therapeutic efficacy is usually constrained by drug resistance, side effects on the body, and tumor recurrence [2]. These challenges highlight the need for novel therapeutics strategies that can effectively control tumor growth with minimal side effects while enhanced chemosensitivity.

Dysregulation of both cell cycle control and apoptosis are hallmark features in GC. Abnormal expressions of Cyclin B1 and CDK1 are key contributors to the uncontrolled transition from the G2 to M phase, driving unchecked cell growth and tumor progression [3]. Simultaneously, the anti-apoptotic mechanism in GC allows tumor cells to resist programmed cell death, adapt to metabolic stress, and develop chemotherapy resistance, thereby promoting cancer progression [4]. The B-cell lymphoma-2 (Bcl-2) protein family is a major regulator of apoptosis, playing a crucial role in the intrinsic mitochondrial apoptosis pathway. This family includes pro-survival proteins (e.g., Bcl-2 and Bcl-xL) and pro-apoptotic proteins (e.g., Bax), which control mitochondrial membrane permeability and cytochrome C release [5]. An increase in Bcl-2 expression disrupts this balance, inhibiting caspase activation and preventing apoptosis, thus promoting tumor cell survival and resistance. BH3-mimetic drugs, such as venetoclax, which mimic the BH3 domain of Bcl-2 to induce apoptosis, have shown strong efficacy in blood-related cancers [6] and solid tumors [7], making Bcl-2 inhibition a promising therapeutic target [8]. In GC, Bcl-2 overexpression is associated with decreased differentiation, lymphatic metastasis, poor prognosis, and resistance to 5-FU [9] and other chemotherapeutic agents [10].

Natural products have long been recognized as a valuable source of anticancer compounds, with an estimated 60% of clinically approved drugs originating from natural sources [11]. The multitarget potential and structural diversity of these compounds often result in potent antitumor activity with low toxicity. *Morus alba* L. *(M. alba*) also produces benzofuran derivatives, such as the Moracin family, which demonstrate anti-apoptotic, anti-inflammatory, and antioxidant properties [12]. Moracin N, for example, inhibits cell division and stimulates apoptosis in non-small cell lung carcinoma by disrupting mitochondria [13]. Moracin D (MD), another bioactive Moracin derivative, has recently gained attention for its anticancer effects. In our previous studies on pancreatic cancer (PC) [14], MD was shown to inhibit cell proliferation, promote apoptosis, and enhance gemcitabine sensitivity via the XIAP/PARP1 pathway. MD also induces apoptosis in breast cancer cells via caspase activation [15] and in prostate carcinoma cells by activating PPAR-γ, modulating anti-apoptotic proteins, and phosphorylating key signaling pathways [16]. However, the impact of MD on GC and its anticancer potential remain underexplored.

In this study, we hypothesized that MD inhibits GC progression by targeting the Bcl-2 pathway, leading to cell-cycle arrest and apoptosis. To evaluate this, we investigated the effects of MD on GC cell viability, proliferation, and colony formation, examining key regulatory proteins such as cyclin B1, CDK1, cleaved PARP, cleaved caspase-3 and -9, and Bcl-2. Additionally, we assessed the functional role of Bcl-2 using lentivirus-mediated overexpression. We also explored the potential synergy between MD and 5-FU, evaluating their combined antitumor efficacy and systemic safety in vitro. Collectively, our findings suggest that MD exerts significant anticancer effects in GC, both in vitro and in vivo, by modulating Bcl-2-mediated apoptosis and inhibiting cell-cycle progression. The observed synergistic effect with 5-FU further supports MD’s potential as a complementary or adjuvant treatment for GC.

## 2. Materials and Methods

### 2.1. Reagents and Antibodies

Moracin D (CAS No. 69120-07-6; HPLC purity ≥ 98%), isolated from *M. alba*, was procured from ChemFaces (Wuhan, China). 5-Fluorouracil (#HY-90006R) was acquired from MedChemExpress (Monmouth Junction, NJ, USA). Dimethyl sulfoxide (DMSO; #D5879), DAPI (#D9542), bromodeoxyuridine (BrdU; #B8434), and MTT reagent (#M5655) were obtained from Sigma-Aldrich (St. Louis, MO, USA). Crystal Violet stain (#C0121), RIPA Lysis Buffer (#P0013B), BCA Kit (#P0012), HRP-anti-mouse (#A0126) and anti-rabbit (#A0208) antibodies, and the Hematoxylin and Eosin (H&E) Staining Kit (#C0105M) were purchased from Beyotime (Shanghai, China). Primary antibodies against cyclin B1 (#55004-1-AP), CDK1 (#19532-1-AP), and α-Tubulin (#80762-1-RR) were procured from Proteintech (Wuhan, China). Antibodies against PARP (#9532T), cleaved-PARP (#5625T), cleaved-caspase-3/-9 (#9664T/#20750), and Bax (#14796T) were sourced from Cell Signaling Technology (Boston, MA, USA). The Bcl-2 antibody (#ab32124) was obtained from Abcam (Cambridge, MA, USA). The antibodies used for Western blot analysis are listed in Supplementary Materials, Table S1.

### 2.2. Cell Culture

The BGC-823, HGC-27, SGC-7901, and MKN-45 human GC cell lines, along with the normal gastric epithelial cell (GES-1), were grown in RPMI-1640 medium (ATCC, Manassas, VA, USA) with 10% FBS and 1% penicillin/streptomycin antibiotic solution. HEK293T cells (ATCC, Manassas, VA, USA) were maintained in DMEM under the same growth conditions. All cells were cultured at 37 °C with 5% CO_2_.

### 2.3. Cell Viability

Cell proliferation and the half-maximal inhibitory concentration (IC_50_) were assessed by culturing cells in 96-well plates at a density of 1 × 10^3^ cells per well. After 24 h of incubation, cells were treated with varying doses of MD for 48 h, with DMSO as the control. In total, 20 μL of MTT solution (5 mg/mL) was pipetted into the wells, followed by a 4 h incubation. The liquid phase was aspirated, 200 μL of control was introduced, and the mixture was determined for absorbance at 540 nm. Cells were exposed to MD at 10, 20, or 40 μM to assess cell growth. Proliferation was then evaluated using the MTT assay on days 1, 3, 5, and 7.

### 2.4. BrdU Staining

GC cells were transferred to the 24-well plates at a concentration of 2 × 10^4^ cells per plate in 500 μL of RPMI-1640 medium and left overnight. MD (20 or 40 μM) was then added to cells, and they were allowed to grow for 24 h in the presence of control as a control. BrdU (5 μg/mL) was then added after treatment and allowed to incubate (2 h) to label proliferating cells. Fixation, antibody incubation, and detection methods were conducted as previously reported [17].

### 2.5. Colony Formation and Soft Agar Assays

For the colony formation assay, GC cells were seeded at 1 × 10^3^ cells per well in 6-well plates and incubated with MD for 14 days. After that, the cells were fixed with 4% paraformaldehyde and subsequently stained with crystal violet (Beyotime, Shanghai, China). Colony formation was analyzed with ImageJ software 1.52a, and the soft agar assay was carried out according to earlier established protocols [14].

### 2.6. Animal Studies and Animal Ethics

The animal experiments were approved by the Southwest University IACUC (IACUC-JFLAB-2025-034, Chongqing, China). Female NOD/SCID mice (4–6 weeks old, Changzhou, China) were housed in an SPF environment. A total of 24 mice were randomly assigned to two groups per cell line (HGC-27 and BGC-823), with 12 per group. For each group, 6 mice were treated with MD (10 mg/kg) and 6 mice with control. HGC-27 or BGC-823 GC cells (1 × 10^6^) were subcutaneously injected into the flanks of the mice to establish xenograft tumors. Upon tumor formation, MD (10 mg/kg) was administered intraperitoneally every 2 days for 18 days, while control animals received an equivalent volume of control. Tumor size and body weight were measured every 3 days, and tumor volume was calculated using the formula: V = (length × width^2^)/2. After 18 days, the mice were euthanized, and tumors were removed, weighed, and photographed for further analysis.

### 2.7. Hematoxylin and Eosin (H&E) Staining

Post-fixation in 4% PFA, tumor tissues were dehydrated and embedded in paraffin. The samples were cut into 3 μm-thick segments. Subsequently, we performed tissue staining and analysis as detailed in the previous study [18].

### 2.8. Immunohistochemistry

Tissue slices were incubated with a 3% oxidizing agent (H_2_O_2_) and then subjected to antigen retrieval using heat-mediated treatment in citrate buffer. Normal serum was applied to block nonspecific binding, followed by overnight incubation with an anti-Ki-67 specific antibody at 4 °C. Afterward, a biotin-conjugated secondary antibody and a streptavidin–peroxidase complex was applied. Immunoexpression was detected using a diaminobenzidine (DAB) substrate solution, and the nuclei were counterstained with hematoxylin. The proportion of Ki-67-positive cells was quantified using a light microscope (Olympus, Japan).

### 2.9. Flow Cytometry Analysis

Flow cytometric analysis of cell apoptosis and the cell cycle was performed by collecting cells treated with MD and assessing them according to the protocol outlined previously [19].

### 2.10. Western Blot (WB) Analysis

After collection and PBS wash, the cell suspensions were lysed on ice in RIPA buffer containing protease inhibitors for 40 min, with intermittent mixing. The resulting lysates were then used for further analyses, as described previously [20].

### 2.11. Molecular Docking Analysis

The structural details of MD were sourced from the PubChem database (https://pubchem.ncbi.nlm.nih.gov/, accessed on 13 November 2025). The Bcl-2 protein’s 3D structure was assessed through UniProt and the RCSB PDB (https://www.rcsb.org/, accessed on 13 November 2025). Molecular docking analyses were performed with AutoDock Vina (Version 1.3.2) (https://vina.scripps.edu/, accessed on 13 November 2025), and the pose with the lowest binding energy was selected as the final result. Both the compound and protein were prepared using AutoDockTools (Version 1.5.7). The 3D structure was visualized using PyMOL (Version 2.3) (https://pymol.org/, accessed on 13 November 2025), and 2D molecular interactions were analyzed by LigPlot+ (https://www.ebi.ac.uk/thornton-srv/software/LigPlus/, accessed on 13 November 2025).

### 2.12. Lentivirus-Mediated Bcl-2 Overexpression

Bcl-2 overexpression and the corresponding empty control plasmids were generated by GeneChem (Shanghai, China). HEK-293T cells were cultured to approximately 90% confluence and co-transfected with the target plasmid and lentiviral packaging plasmids using the Hieff Trans™ lipid-based transfection reagent (Yeasen, China). After 48 h, viral supernatants were collected, filtered through a 0.45-μm membrane, and used to transduce GC cells (approximately 30–40% confluence) with polybrene (Sigma, USA) for 24 h. The infection was repeated to enhance efficiency. Stable transductants were selected using puromycin (2 μg/mL) for 2 weeks, and Bcl-2 overexpression was confirmed by WB analysis.

### 2.13. Combination Treatment Assay

To assess the combined effect of MD and 5-FU, GC cells were exposed to MD (10 μM), 5-FU (5 μM), or both drugs within 48 h. The cell growth was measured using MTT and BrdU incorporation assays. The Jin formula was used to determine synergism:

$$Q=\frac{E\left(A+B\right)}{\left(EA+EB-EA*EB\right)}$$

where E (A + B) refers to the combined inhibition from the two treatments, and EA and EB indicate the effects of the individual agents. A Q value > 1.15 indicates synergism, 0.85–1.15 indicates an additive effect, and <0.85 indicates antagonism.

### 2.14. Statistical Analysis

Data were processed using GraphPad Prism 10.1.2 and are presented as mean ± SD. Quantification was performed using ImageJ software 1.52a, and values are expressed as the ratio of the target protein to the loading control. All experiments were conducted in triplicate, and statistical significance was assessed using Student’s *t*-test and one-way ANOVA. *p*-values are indicated as follows: * *p* < 0.05, ** *p* < 0.01, and *** *p* < 0.001.

## 3. Results

### 3.1. Moracin D Inhibits GC Cell Proliferation

**Figure 1:**
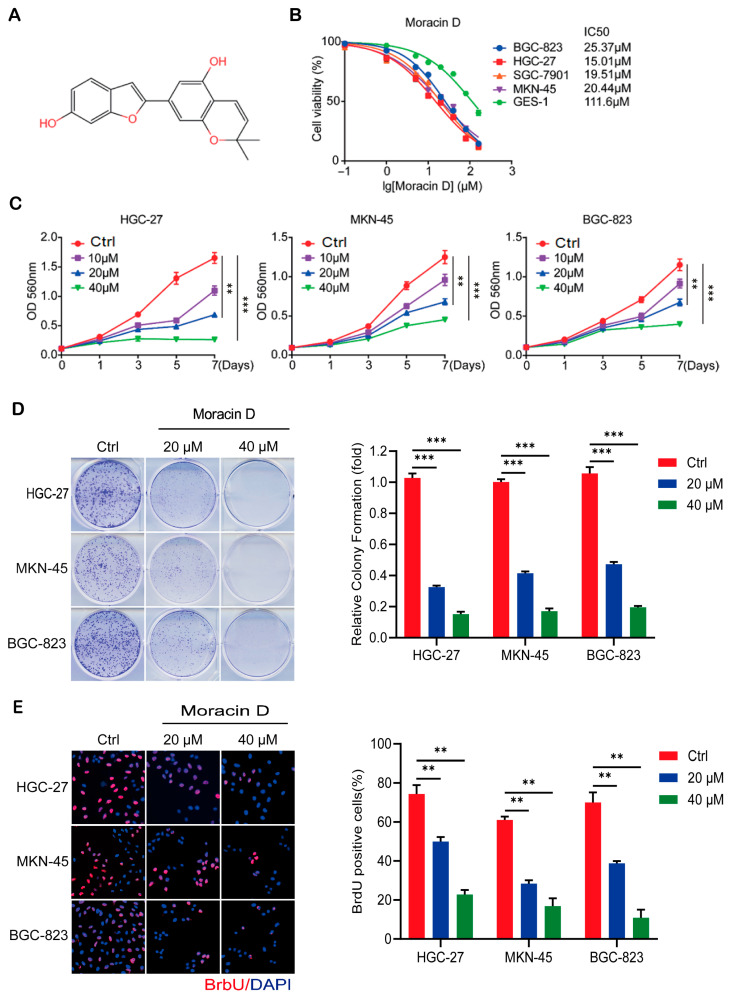
Moracin D suppresses viability and proliferation of GC cells. (**A**) Molecular configuration of MD. (**B**) GC cells and normal GES-1 cells were cultured with varying concentrations of MD for 48 h, and the IC_50_ values were determined by the MTT assay, with control (Ctrl) treatment as a baseline. (**C**) Cell viability of GC cells was assessed after treatment with MD (10, 20, and 40 μM) for 1–7 days. (**D**) Colony formation assay showing reduced colony counts and smaller colony sizes in GC cells treated with MD (20 μM and 40 μM) after 14 days. (**E**) BrdU incorporation assay in GC cells following 48 h of incubation with MD (20 μM, 40 μM). Representative images of BrdU-positive cells are shown on the left, with quantification on the right. Scale bar = 40 μm. All data are presented as the means ± S.D., ** *p* < 0.01, *** *p* < 0.001.

In our lab’s previous research, we screened the chemical compounds of *M. alba* and its derivatives using the TCMSP database, where MD was identified across several databases. KEGG pathway enrichment analysis of the identified targets revealed strong associations with cancer-related signaling pathways, further supporting its anti-tumor activity [14]. Among these bioactive compounds, MD (chemical structure presented in Figure 1A) was particularly highlighted. To assess the anti-cancer efficacy of MD, gastric cancer cell lines were treated with varying concentrations of MD for 48 h, with the control. MTT analysis revealed IC_50_ values of 25.37 μM, 15.01 μM, 19.51 μM, and 20.44 μM in BGC-823, HGC-27, SGC-7901, and MKN-45 cells, respectively (Figure 1B). In contrast, the IC_50_ for MD in normal mucosal epithelial cells (GES-1) was 111.6 μM, which is higher than the values in the four gastric cancer cell lines (Figure 1B), indicating that MD selectively targets carcinoma cells. This suggests that GC cells are more sensitive to MD treatment than GES-1 cells. SGC-7901 was excluded from most experiments due to inconsistent growth and proliferation, likely resulting from variability in cell culture conditions. To ensure reliability, we focused on BGC-823, HGC-27, and MKN-45, which showed consistent responses to MD. The MTT assay further confirmed that MD significantly inhibited cell proliferation in a dose-dependent manner (Figure 1C). Similarly, in vitro colony formation assays revealed a significant decrease in both the number and size of colonies following MD treatment (Figure 1D). BrdU incorporation assays also showed a marked decline in the percentage of BrdU-positive GC cells (Figure 1E). Together, these results highlight the potent inhibitory effects of MD on DNA synthesis and overall proliferation in GC cells.

### 3.2. Moracin D Inhibits Clonogenicity and Tumorigenesis in GC Cells

**Figure 2:**
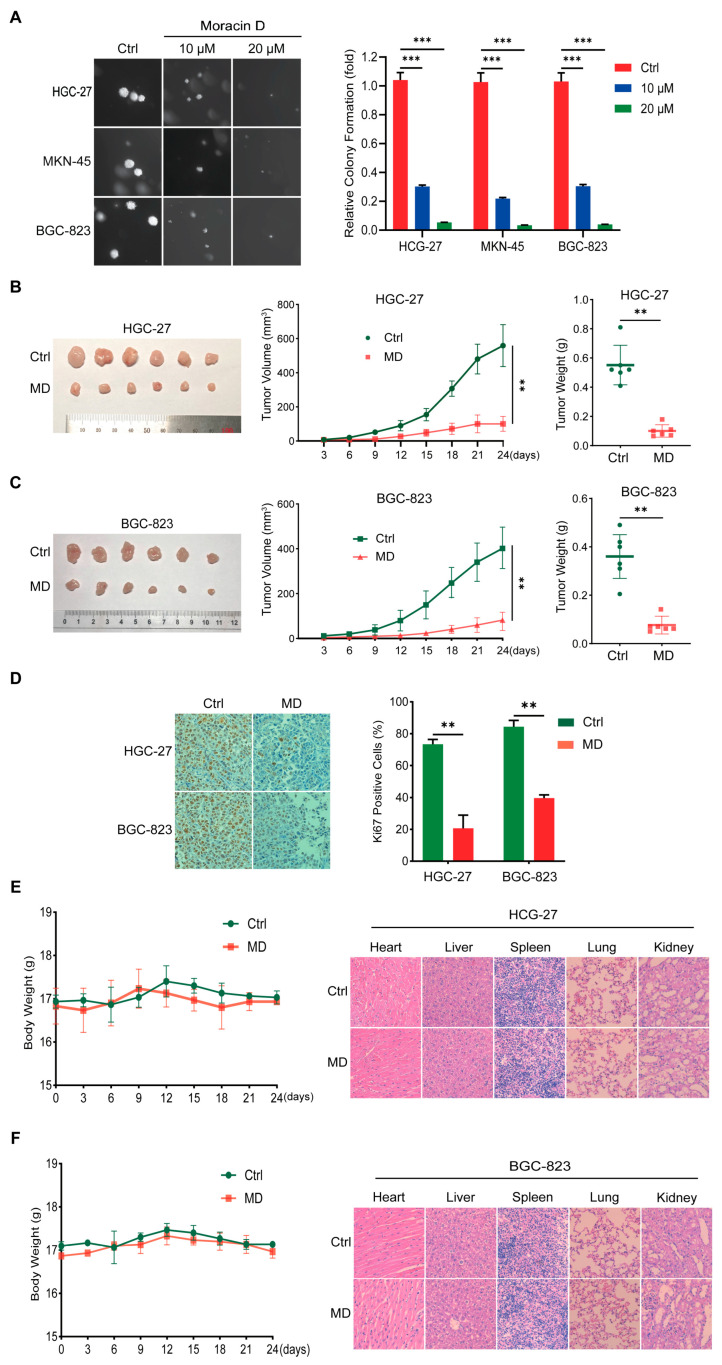
Moracin D suppresses clonogenicity and tumorigenesis in GC cells. (**A**) Soft agar assay of GC cells treated with MD (10 and 20 μM) or control for 3 weeks. Colony images and quantification are shown. Scale bar = 300 μm. (**B**,**C**) Tumor xenograft assays using HGC-27 (**B**) and BGC-823 (**C**) cells, with treatment of MD (10 mg/kg). Representative images of tumors (**left**), tumor volume growth curves (**center**), and tumor weights (**right**) are shown (n = 6 per group). (**D**) Immunohistochemical staining for Ki-67 (**left**) and quantification of Ki-67-positive cells (**right**) in tumors from HGC-27 and BGC-823 xenograft mice treated with MD or control. Scale bar = 100 μm. (**E**,**F**) Body weight and organ histology of mice: (**left** side) body weight remained stable following subcutaneous transplantation of HGC-27 (**E**) and BGC-823 (**F**) cells; (**right** side) H&E staining of key organs showed no noticeable morphological changes in mice bearing HGC-27 (**E**) and BGC-823 (**F**) tumors. Scale bar = 100 μm. Data are presented as means ± S.D., ** *p* < 0.01, *** *p* < 0.001.

To evaluate the antitumor effect of MD, we first assessed its impact on the GC cell clonogenicity using a soft agar assay. Treatment with MD significantly reduced both the number and size of colonies formed by GC cells compared to the control group (Figure 2A), suggesting that MD effectively inhibits the clonogenic expansion of GC cells in vitro.

The effects of MD on HGC-27 and BGC-823 cells in vivo were evaluated by injecting the cells subcutaneously into nude mice at 4-week intervals, with tumor growth monitored every 2 days. Tumor progression was significantly suppressed in the MD-treated group compared to the control. Tumor volume and weight were notably reduced in MD-treated mice (Figure 2B,C). Immunohistochemical staining further confirmed a significant reduction in Ki-67 expression in tumor samples from the MD-treated group (Figure 2D), supporting the notion that MD inhibits GC cell proliferation and tumorigenesis in vivo. Importantly, no obvious toxicity was observed in MD-treated mice. Body weight remained stable throughout the experimental period (Figure 2E,F, left), and histopathological analysis with H&E staining showed no significant morphological abnormalities (Figure 2E,F, right). Collectively, these findings suggest that MD effectively inhibits the clonogenic and tumorigenic abilities of GC cells, without inducing systemic or organ toxicity, highlighting its potential as a safe and effective therapeutic option for GC.

### 3.3. Moracin D Induces Cell Cycle Arrest at the G2/M Phase in GC Cells

**Figure 3:**
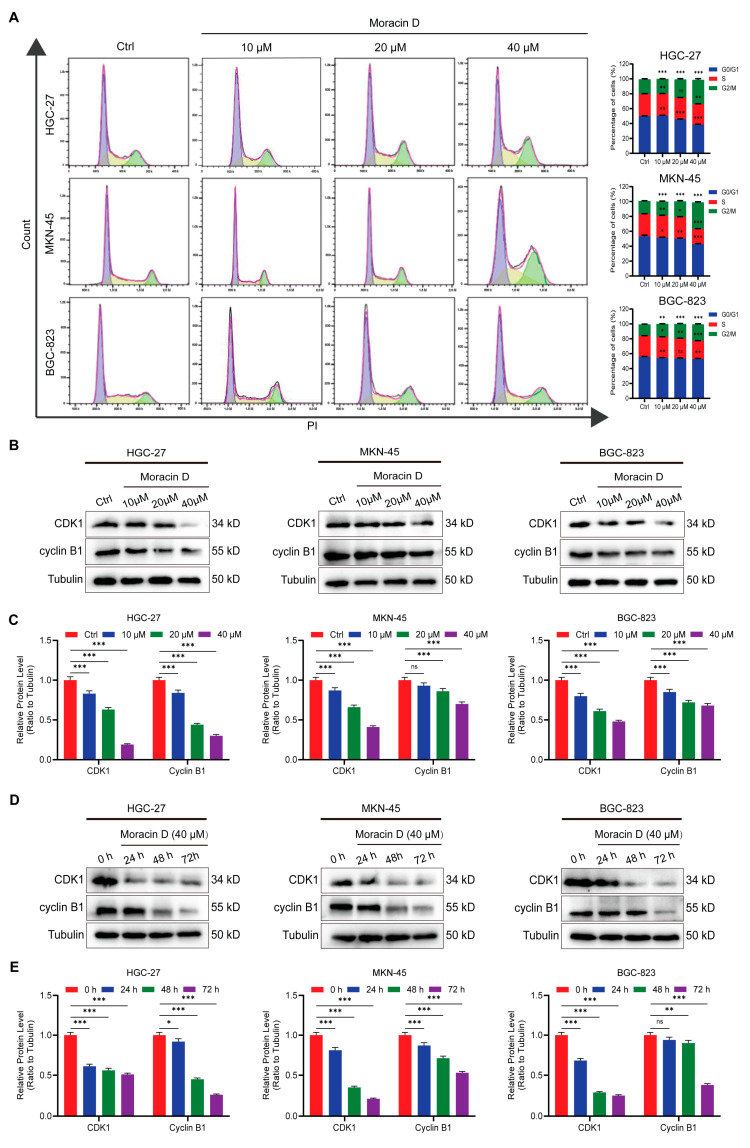
Moracin D induces G2/M phase cell cycle arrest in GC cells. (**A**) Flow cytometry analysis of the cell cycle in GC cells treated with MD (10, 20 or 40 μM) for 48 h (**left**), with quantification of cells in each phase shown (on the **right**). (**B**) Western blot analysis of Cyclin B1 and CDK1 expression in GC cells exposed to MD (10, 20, or 40 μM) or control for 48 h. (**C**) Densitometric quantification of the WB results from panel B. (**D**) Time-dependent changes in Cyclin B1 and CDK1 expression in GC cells treated with 40 μM MD for 0, 24, 48, and 72 h. (**E**) Densitometric quantification of the WB results from panel D. Tubulin was used as the loading control. The original WB images are shown in Figure S1. All data are presented as the means ± S.D., * *p* < 0.05, ** *p* < 0.01, *** *p* < 0.001. ns: not significant.

Uncontrolled proliferation is a hallmark of cancer, often resulting from dysregulation of the cell cycle. To investigate whether MD affects cell cycle progression in GC, flow cytometry analysis was performed on GC cells treated with 10 μM, 20 μM or 40 μM MD for 48 h. MD treatment caused a significant increase in the percentage of cells in the G2/M phase, accompanied by a concurrent decrease in the G0/G1 and S phase populations (*p* < 0.05). This dose-dependent response suggests that MD effectively inhibits cell cycle progression by inducing G2/M arrest in GC cells (Figure 3A).

To further investigate the underlying mechanism, we examined the levels of major regulators of the G2/M checkpoint. WB experiments confirmed that MD treatment induced a significant, dose-dependent decrease in CDK1 and cyclin B1 protein levels across all three cell lines (Figure 3B,C). Additionally, time-dependent experiments revealed that prolonged exposure to MD (0, 24, 48, and 72 h) progressively reduced CDK1 and cyclin B1 expression (Figure 3D,E), indicating a sustained inhibitory effect on mitotic entry. Collectively, these results demonstrate that MD inhibits GC cell growth by suppressing the CDK1/cyclin B1 signaling axis, leading to G2/M phase arrest and impaired mitotic progression. This mechanism highlights the potential of MD as a promising cell cycle-targeted therapeutic agent for GC.

### 3.4. Moracin D Induces Apoptosis in GC Cells

**Figure 4:**
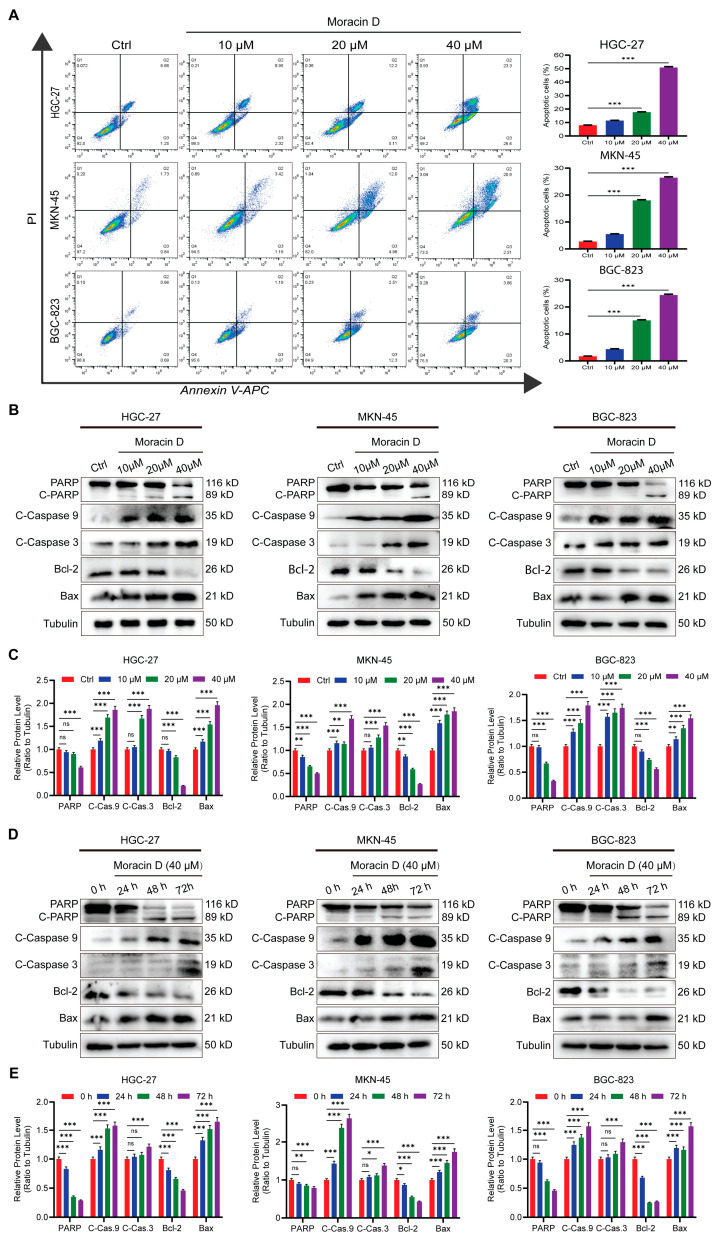
Moracin D triggers apoptosis in GC cells. (**A**) Flow cytometry was used to quantify the rate of apoptosis in GC cells treated with MD (10, 20 and 40 μM) for 48 h, with control cells as a baseline. Flow cytometry plots and corresponding quantitative analyses are presented. (**B**) Western blot analysis of apoptotic markers in GC cells exposed to MD (10, 20, and 40 μM) or control for 48 h. Levels of Bcl-2, Bax, PARP, C-PARP, and C-caspase-3/-9 were measured, with Tubulin as a loading control. (**C**) Densitometric quantification of the WB results from panel B. (**D**) Time-dependent changes in apoptosis-related proteins of Bcl-2, Bax, C-PARP, and C-caspase-3/-9 in GC cells treated with 40 μM MD for 0, 24, 48, and 72 h. (**E**) Densitometric quantification of the WB results from panel D. The original WB images are shown in Figures S2 and S3. All data are presented as the means ± S.D., * *p* < 0.05, ** *p* < 0.01, *** *p* < 0.001. ns: not significant.

Numerous anti-cancer agents exert their effects by triggering apoptosis in tumor cells. To assess whether MD induces apoptosis in GC cells, the cells were exposed to 10 μM, 20 μM and 40 μM MD for 48 h. The apoptosis rate was evaluated by flow cytometry using Annexin V-APC/PI staining. MD treatment significantly increased the proportion of apoptotic cells compared to controls, with the 40 μM group showing the highest apoptotic rate (Figure 4A). Additionally, Western blot analysis was conducted to validate these findings. Cells were treated with 10, 20, and 40 μM MD for 48 h, and the levels of major apoptosis-related proteins were measured. The apoptosis-regulating proteins Bcl-2 and PARP were downregulated by MD treatment in a dose-dependent manner. In contrast, cleaved PARP (C-PARP), cleaved caspases-3/-9 (C-caspase-3/-9), and the apoptosis-inducing protein Bax were significantly upregulated (Figure 4B,C). Time-dependent experiments (0, 24, 48, and 72 h) further revealed that Bcl-2 and PARP levels progressively decreased with prolonged exposure to 40 μM MD, while C-PARP, C-caspase-3/-9, and Bax levels continued to increase (Figure 4D,E). Tubulin was used as the control in all WB analyses. Overall, these findings suggest that MD induces apoptosis in GC cells in a dose- and time-dependent manner, primarily by activating the intrinsic mitochondrial apoptotic pathway and modulating Bcl-2 family proteins.

### 3.5. Overexpression Bcl-2 Attenuate Moracin D-Induced Inhibition of GC Cell Proliferation

**Figure 5:**
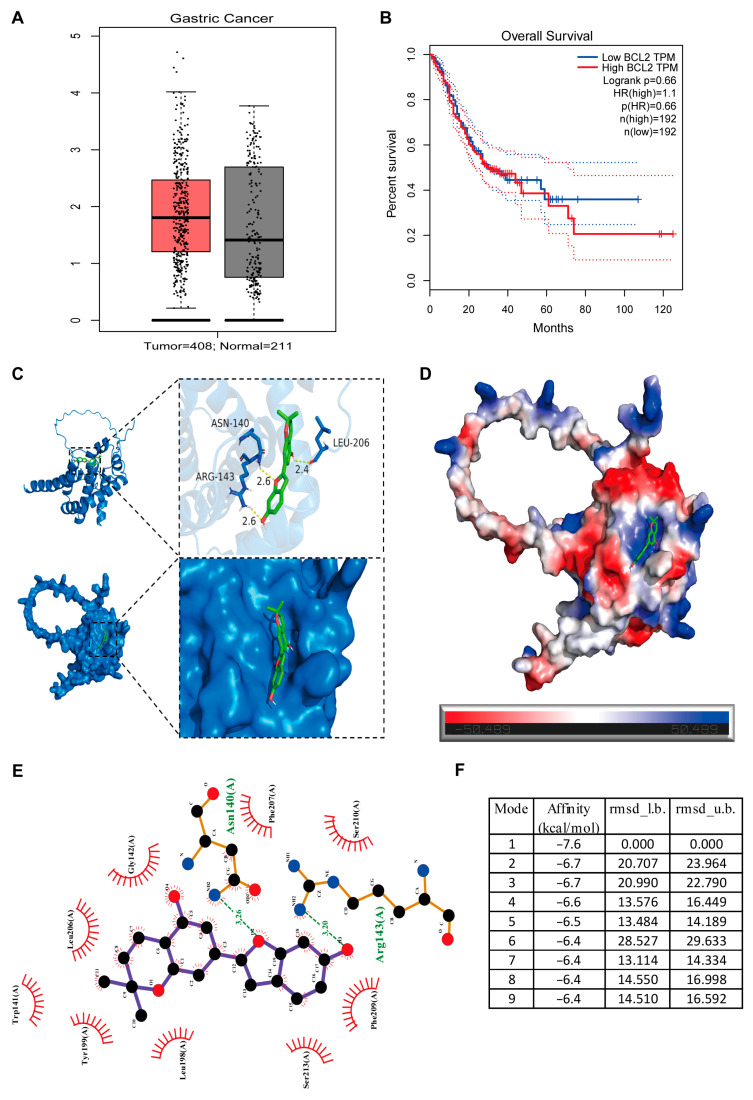
Moracin D directly binds to BCL2. (**A**) Bcl-2 expression in GC and normal tissues, as retrieved from the GEPIA database. (**B**) The GEPIA database shows a strong correlation between up-regulated Bcl-2 expression and unfavorable prognosis in GC. (**C**) Three-dimensional docking model of MD, demonstrating its binding to the active site of the Bcl-2 protein. (**D**) Electrostatic surface interactions between MD and Bcl-2, highlighting key binding features. (**E**) Two-dimensional representation of the MD-Bcl-2 complex, illustrating the molecular interaction. (**F**) Docking analysis showing the lowest binding energy configuration for the interaction between MD and Bcl-2.

**Figure 6:**
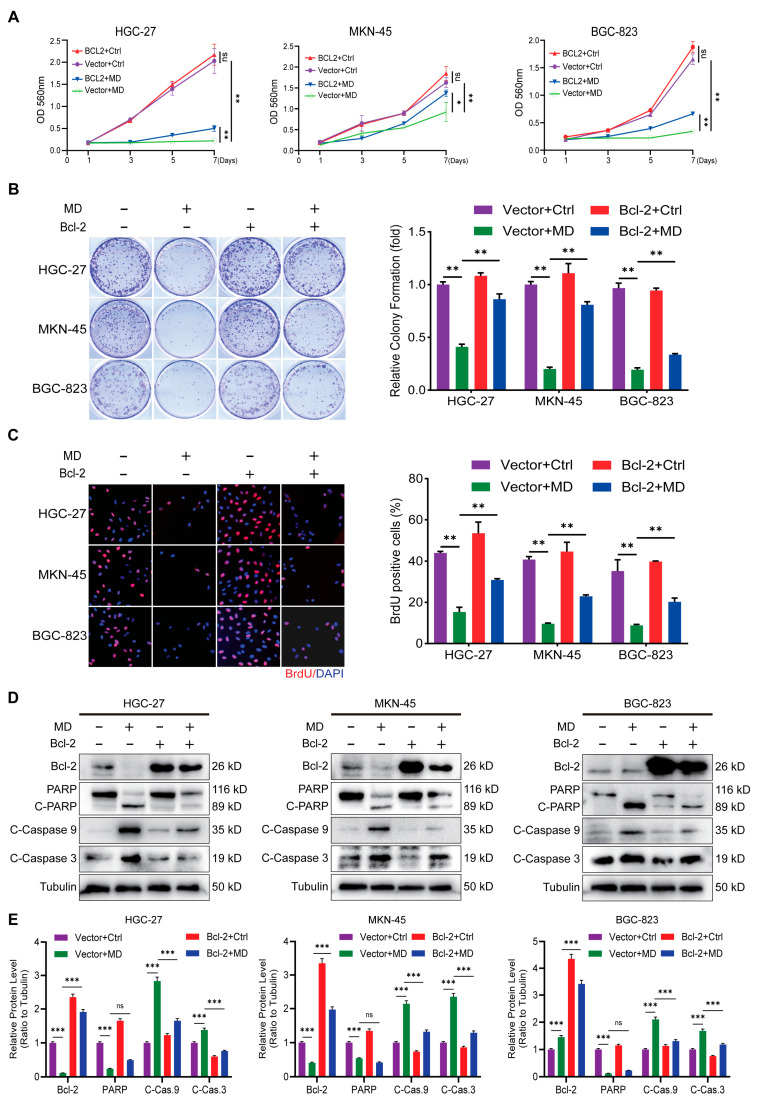
Bcl-2 overexpression reduces the proliferation-suppressing and apoptosis-inducing effects of MD in GC cells. (**A**) MTT assay measuring cell proliferation in vector- or Bcl-2–overexpressing cells treated with MD (20 μM) or control for 1–7 days. (**B**) Colony formation assay in vector- or Bcl-2–overexpressing cells incubated with MD (20 μM) or control for 14 days. Images are shown on the left, with quantified colony numbers on the right. (**C**) BrdU incorporation assay evaluating DNA synthesis in vector- or Bcl-2–overexpressing cells exposed to MD (20 μM) or control. Images (**left**) and quantification of BrdU-positive cells (**right**) are presented. Scale bar = 100 μm. (**D**) Western blot analysis of apoptotic proteins in vector- or Bcl-2–overexpressing GC cells treated with MD (20 μM) or control for 48 h. (**E**) Densitometric quantification of the WB results from panel D. The original WB images are shown in Figure S4. All data are presented as the means ± S.D., * *p* < 0.05, ** *p* < 0.01, *** *p* < 0.001. ns: non signigicant.

Bcl-2 is a crucial apoptosis inhibitor that is often aberrantly expressed in cancer cells [21,22]. According to data from the GEPIA database (http://gepia.cancer-pku.cn/, accessed on 19 December 2025), Bcl-2 levels are significantly elevated in GC tissues compared to normal tissues (Figure 5A), and higher Bcl-2 levels are associated with poorer overall survival (Figure 5B). Molecular docking simulations revealed that MD binds strongly to the Bcl-2 active site with higher affinity (Figure 5C–F). The Bcl-2 active site interacts with MD, exhibiting the lowest affinity and most suitable 3D docking structure (Figure 5C,D). In this interaction, electrostatic and hydrophobic forces form nonbonded contacts, suggesting the formation of a stable, strong complex (Figure 5D,E). The most favorable docking results (lowest binding energies) were below −7 kcal/mol (Figure 5F), supporting the hypothesis that Bcl-2 may be a key molecular target of MD. Based on these results, we hypothesize that Bcl-2 is a target of MD in mediating its anti-proliferative and pro-apoptotic effects in GC. To investigate this, we generated stable Bcl-2–overexpressing cell lines with empty vector controls. MTT assays demonstrated that Bcl-2 overexpression rescued cell viability following MD treatment (Figure 6A). Similarly, colony formation assays demonstrated partial restoration of clonogenic capacity (Figure 6B), and BrdU incorporation assays confirmed the recovery of DNA synthesis (Figure 6C). Western blot analysis showed that Bcl-2 overexpression attenuated MD-induced cleavage of caspase-3 and -9, but did not affect PARP expression (Figure 6D,E). Collectively, these findings underscore that Bcl-2 is an important mediator of MD-induced growth inhibition and apoptosis.

### 3.6. Moracin D Enhances Chemosensitivity of GC Cells to 5-Fluorouracil

**Figure 7:**
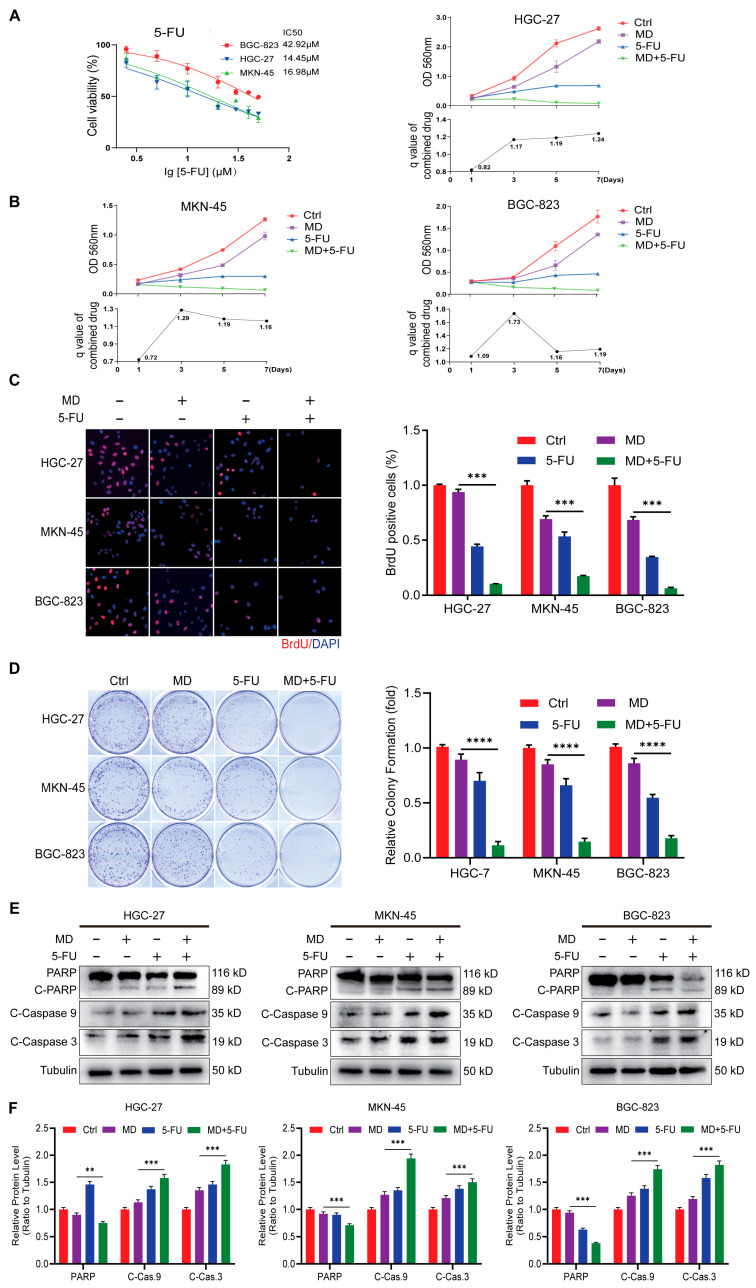
Moracin D enhances GC cell chemosensitivity to 5-Fluorouracil. (**A**,**B**) MTT assays were used to assess cell viability in GC cells treated with MD (10 μM) and 5-FU (5 μM), or co-treated with both agents (10 μM MD + 5 μM 5-FU) for 1–7 days. Control-treated cells served as the baseline. The combination index (q) was calculated using Jin’s formula to evaluate the synergistic interaction between MD and 5-FU. (**C**) BrdU incorporation assay to evaluate DNA synthesis following treatment with MD and/or 5-FU. Representative images (**left**; scale bar = 100 μm) are shown, with the proportion of BrdU-positive cells quantified (on the **right**). (**D**) Clonogenic assay to assess colony formation following treatment with MD and 5-FU, with relative colony formation quantified. (**E**) Western blot analysis of apoptotic proteins in GC cells treated with MD (10 μM), 5-FU (5 μM), or their combination for 48 h. (**F**) Densitometric quantification of the WB results from panel D. The original WB images are shown in Figure S5. All data are presented as the means ± S.D., ** *p* < 0.01, *** *p* < 0.001, **** p < 0.0001.

5-FU is a well-established chemotherapeutic agent used in the treatment of GC, but both endogenous and exogenous resistance often limit its therapeutic effectiveness. To date, no studies have explored the combined effects of 5-FU and MD in GC. Using Jin’s formula with *q*-values ≥ 1.15, we observed a synergistic effect of 5-FU and MD across various concentrations and treatment durations. To further investigate whether MD enhances the antitumor effects of 5-FU, GC cells were treated with 5-FU alone or in combination with 10 μM MD for 48 h. The IC_50_ concentrations of 5-FU in BGC-823, HGC-27, and MKN-45 cells were 42.92 μM, 14.45 μM, and 16.98 μM, respectively. The 10 µM MD concentration was selected for combination therapy with 5-FU, based on previous in vitro and in vivo results demonstrating significant anti-cancer efficacy and good tolerability. This dose effectively balanced efficacy and minimized toxicity, and it was consistent with the 10 mg/kg MD dose used in our in vivo experiments. According to MTT assays, the combination of MD and 5-FU had a greater impact on growth inhibition than when used separately, indicating a synergistic effect (Figure 7A,B). Similarly, the BrdU assay showed that the MD-5-FU combination was significantly more effective at suppressing DNA synthesis than single-agent treatments (Figure 7C). The clonogenic assay also demonstrated that the co-treatment with 5-FU and MD markedly decreased cell proliferation compared to the individual treatments (Figure 7D). Furthermore, WB showed that the synergistic treatment significantly increased Bax expression and the cleavage of apoptosis-related proteins, including PARP and caspase-3/-9, while decreasing Bcl-2 and PARP levels (Figure 7E,F and Figure S6A,B). Altogether, these results suggest that MD substantially enhances the response of GC cells to 5-FU by inducing apoptotic signaling and inhibiting proliferation, highlighting its potential as an adjuvant in 5-FU-based chemotherapy.

## 4. Discussion

Gastric cancer is a major contributor to cancer-related mortality, presenting a significant therapeutic challenge due to its aggressive nature and the lack of effective treatment options. Conventional therapies for GC mainly involve surgery, chemotherapy, and radiation, but their effectiveness is often limited by resistance and cytotoxic effects [23,24]. Consequently, there is growing interest in bioactive natural substances that may selectively inhibit tumor growth and enhance therapeutic outcomes, offering a potential solution to the limitations of traditional treatments [25,26]. Flavonoids, a class of plant-derived compounds, show considerable promise for cancer treatment, particularly in various cancer types. Morus alba (*M. alba*), a plant rich in bioactive compounds, has demonstrated significant anti-cancer potential, with compounds such as Kuwanon-A [27,28], Morusin [29], Sanggenon C, and MD showing promising outcomes in various studies [30]. In our study, MD appears to act as a potent and selective inhibitor of GC progression. Testing across multiple human GC cell lines (BGC-823, HGC-27, and MKN-45) revealed that MD significantly suppressed cell survival in a dose- and time-dependent manner. Notably, MD displayed strong tumor selectivity, with lower IC_50_ values in GC cells compared to normal gastric cells, and even the highest MD concentration showed no detectable toxicity in normal gastric cells.

Functional assays further support MD’s anti-proliferative effects, showing a significant reduction in cell proliferation. Mechanistically, colony formation was notably impaired following treatment, with both colony number and size significantly reduced. Soft agar assays revealed decreased anchorage-independent growth, and BrdU incorporation analyses confirmed a decline in DNA synthesis in MD-treated cells. In vivo xenograft experiments generally supported the in vitro findings; MD inhibited tumor growth without significant adverse effects on body weight or organ damage, suggesting potent anti-tumor effects with a potentially favorable safety profile. Collectively, these findings suggest that MD could effectively suppress GC cell growth in vitro and inhibit tumor development in vivo, though further studies are needed to confirm these effects in a clinical context.

The cell cycle is fundamentally linked to cell proliferation, and its dysregulation is a hallmark of cancer, driving uncontrolled cell division and genomic instability [31]. Crucial regulators, such as cyclins, cyclin-dependent kinases (CDKs), and cell cycle checkpoint proteins, coordinate the progression of the cell cycle, and their altered expression has been implicated in tumorigenesis [32,33]. We found that MD exhibits broad-spectrum antitumor activity in GC cells by mediating G_2_/M phase cell-cycle arrest in a dose- and time-dependent manner. These findings align with previous studies showing that mulberry compounds, such as demethylzeylasteral (DEM), induce G_2_/M arrest by suppressing Cyclin B1 and CDK1 [34], and that deoxyelephantopin (DET) induces a similar arrest in colon cancer [35]. In line with these observations, our study suggests that reductions in Cyclin B1 and CDK1 may indicate that MD disrupts mitotic progression, preventing aberrant cell division [36,37].

Moracin D has been shown to induce apoptosis in various tumors, including breast [15], prostate [16], and pancreatic cancer [14]. Given that the failure of malignant cells to undergo apoptosis promotes tumor development and treatment resistance [5], we performed an apoptosis assay and WB analysis to determine whether MD could also mediate cell death in GC cells. Our experimental findings revealed that MD triggers apoptosis in GC cells by activating the intrinsic mitochondrial apoptotic pathway. This was evidenced by the activation of Bax, cleavage of caspases-3/-9 and PARP, and reduced Bcl-2 expression, which modulate mitochondrial permeability, restrict cytochrome c release, and inhibit caspase activation [38,39,40]. The suppression of Bcl-2 by MD could destabilize mitochondrial integrity and amplify the caspase-dependent apoptotic cascade. Together, these findings suggest that MD may arrest the cell cycle and reactivate Bcl-2-mediated apoptosis, potentially sensitizing GC cells to regulated cell death, although further validation is necessary to fully understand the underlying mechanisms.

Moreover, our data suggest that Bcl-2 expression plays a key role in GC prognosis. We found that dysregulated Bcl-2 expression correlates with poor overall survival (OS) in GC patients, consistent with previous studies [21,41]. To validate the functional role of Bcl-2, we conducted molecular docking simulations, which indicated that MD binds strongly to the Bcl-2 active site, suggesting that Bcl-2 could be a direct target of MD. We successfully overexpressed Bcl-2 in GC cells using lentiviral transfection and confirmed its expression via immunoblotting. The phenotypic effects induced by MD were partially reversed in these Bcl-2-overexpressing cells, as indicated by restored cell proliferation, increased clonogenic potential, and reduced apoptosis. This reversal emphasizes the critical role of Bcl-2 suppression in MD-induced cytotoxicity. Our findings suggest that Bcl-2 appears to be an important mediator of Moracin D’s anti-cancer effects, making it a critical target for the compound’s ability to inhibit GC cell growth and trigger apoptosis. These results are consistent with earlier studies showing that natural compounds can promote apoptosis by modulating Bcl-2 [42,43].

5-FU suppresses thymidylate synthase, blocking nucleotide synthesis and triggering DNA damage [44]. However, resistance to 5-FU often develops in malignant cells due to activation of DNA damage repair pathways and enhanced anti-apoptotic mechanisms, limiting its therapeutic efficacy [45]. To overcome this challenge, combination therapy is a promising strategy to augment chemotherapy effectiveness while alleviating toxicities [46]. In our study, we found that co-treatment with MD significantly reduced the 5-FU IC_50_, enhanced the cleavage of apoptotic proteins, and suppressed DNA synthesis, indicating a potential synergistic interaction. This enhancement seems to result from MD-mediated suppression of Bcl-2, which could lower the apoptotic threshold and allow 5-FU-induced DNA damage to more effectively trigger caspase activation. These results support previous research showing that natural compounds, such as DEM and curcumin, can potentiate 5-FU activity by targeting oncogenic and survival signaling pathways [47,48].

**Figure 8:**
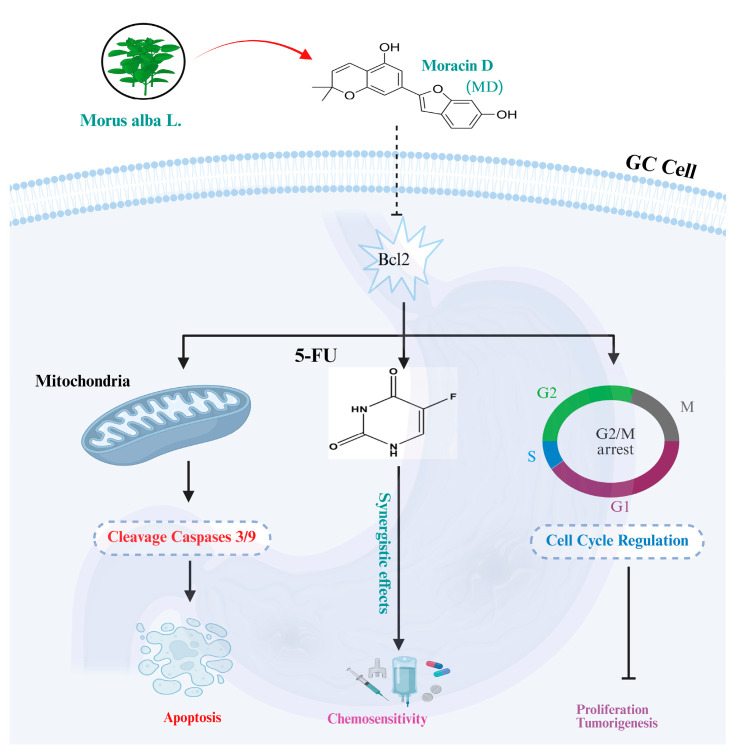
Illustration of the molecular mechanism triggered by MD in BGC-823, HGC-27, and MKN-45 GC cells.

Taken together, our study suggests that MD’s ability to induce G_2_/M arrest, activate mitochondrial apoptosis, and enhance cellular responsiveness to 5-FU makes it a promising dual-acting agent to address key mechanisms of chemoresistance. Notably, MD exhibited minimal toxicity to normal GES-1 cells and in vivo models, highlighting its favorable safety profile. Furthermore, our studies provide preliminary preclinical evidence suggesting that moracin D affects Bcl-2-related apoptotic pathways and may potentially enhance the response of gastric cancer cells to 5-FU treatment. MD targets cell-cycle regulators and apoptosis pathways, directly addressing two key hallmarks of therapeutic resistance: uncontrolled proliferation and evasion of apoptosis. Due to this dual-target mechanism, the therapy appears to have greater effectiveness, with significantly improved patient outcomes, making MD a highly promising therapeutic option. Moreover, these results emphasize the potential of MD, either as a monotherapy or in combination with 5-FU, to reduce the dosages of standard cytotoxic regimens and minimize their side effects, as illustrated in Figure 8. This strategy may enhance therapeutic efficacy and patient safety by reducing the risk of toxicity. However, further research is needed to fully understand MD’s therapeuti**c** mechanisms, its synergistic interaction with Bcl-2, and the transcriptomic changes involved. Preclinical trials are also crucial for validating the synergistic effects of MD and 5-FU and optimizing dosing strategies to improve efficacy and safety. These efforts are vital for translating MD from preclinical models to clinical applications in the treatment of gastric cancer.

In summary, our study showed that MD, a flavonoid derived from *M. alba*, can effectively inhibit GC progression by inducing G_2_/M cell cycle arrest and triggering mitochondrial apoptosis. MD’s dual-target mechanism involves suppressing the Bcl-2 signaling pathway, leading to destabilization of mitochondria and activating the apoptotic process. In vitro, MD reduced GC cell proliferation, colony formation, and DNA synthesis while promoting cell death. It also improved the effectiveness of 5-FU by enhancing DNA damage and apoptosis in GC cells. Importantly, under the experimental conditions used, MD did not show any clear cytotoxicity towards normal gastric cells or obvious signs of toxicity in in vivo models. These results provide preclinical observations suggesting that MD may exhibit antitumor activity in gastric cancer models, both as monotherapy and in combination with 5-FU.

## 5. Conclusions

MD demonstrates promising anticancer activity in GC by targeting the Bcl-2-mediated cell cycle and apoptotic pathway which may contribute to enhancing the effects of chemotherapy. The observed synergistic interaction between MD and 5-FU suggests that MD could serve as an adjunct to standard GC treatments, potentially reducing the toxicity of chemotherapeutic regimens while improving treatment outcomes. Given its dual-target mechanism, favorable safety profile, and the encouraging preclinical data, MD shows potential for further investigation in clinical applications for GC. However, additional studies are required to better understand the mechanisms through which MD modulates Bcl-2 and its effects on other molecular pathways involved in GC progression. Preclinical trials and clinical validation will be crucial for refining MD’s dosing strategy and determining its role in GC treatment.

## Data Availability

The data that support the findings of this study are available from the corresponding author upon reasonable request.

## Supplementary Materials

The following supporting information can be downloaded at: https://www.mdpi.com/article/10.3390/biom16030428/s1, Figure S1: The original WB images of Figure 3B,D; Figure S2: The original WB images of Figure 4B; Figure S3: The original WB images of Figure 4D; Figure S4: The original WB images of Figure 6D; Figure S5: The original WB images of Figure 7E; Figure S6: Bcl-2 and Bax protein expression in GC cells; Table S1. Antibodies information used for WB.

## Abbreviations

The following abbreviations are used in this manuscript:

GC	Gastric cancer
MD	Moracin D
Bcl-2	B-cell lymphoma-2
5-FU	5-fluorouracil
*M. alba*	*Morus alba* L.
PC	Pancreatic cancer
BC	Breast cancer
DMSO	Dimethyl sulfoxide
BrdU	Bromodeoxyuridine
H&E	Hematoxylin and eosin
DAB	Diaminobenzidine
WB	Western blot
GES-1	Gastric mucosal epithelial cells
CDKs	Cyclin-dependent kinases
DET	Deoxyelephantopin
OS	Overall survival
DEM	Demethylzeylasteral

## References

[1] Bray F., Laversanne M., Sung H., Ferlay J., Siegel R.L., Soerjomataram I., Jemal A. (2024). Global cancer statistics 2022: GLOBOCAN estimates of incidence and mortality worldwide for 36 cancers in 185 countries. CA Cancer J. Clin..

[2] Joshi S.S., Badgwell B.D. (2021). Current treatment and recent progress in gastric cancer. CA Cancer J. Clin..

[3] Javed A., Yarmohammadi M., Korkmaz K.S., Rubio-Tomás T. (2023). The Regulation of Cyclins and Cyclin-Dependent Kinases in the Development of Gastric Cancer. Int. J. Mol. Sci..

[4] Hanahan D. (2022). Hallmarks of Cancer: New Dimensions. Cancer Discov..

[5] Qian S., Wei Z., Yang W., Huang J., Yang Y., Wang J. (2022). The role of BCL-2 family proteins in regulating apoptosis and cancer therapy. Front. Oncol..

[6] Anderson M.A., Deng J., Seymour J.F., Tam C., Kim S.Y., Fein J., Yu L., Brown J.R., Westerman D., Si E.G. (2016). The BCL2 selective inhibitor venetoclax induces rapid onset apoptosis of CLL cells in patients via a TP53-independent mechanism. Blood.

[7] Wang J.Q., Li J.Y., Teng Q.X., Lei Z.N., Ji N., Cui Q., Zeng L., Pan Y., Yang D.H., Chen Z.S. (2020). Venetoclax, a BCL-2 Inhibitor, Enhances the Efficacy of Chemotherapeutic Agents in Wild-Type ABCG2-Overexpression-Mediated MDR Cancer Cells. Cancers.

[8] Alharbi F., Almanifi E., Ashrafuzzaman M. (2024). Targeting BCL-2 family proteins using BH3 mimetic drugs for cancer therapy: A systematic review of randomized clinical trials. Med. Drug Discov..

[9] Cheng H., Wang X., Li T., Chen L. (2015). Bcl-2 expression and patient survival in gastric cancer: A systematic review of the literature with meta-analysis. Med. Oncol..

[10] Soni S., Anang V., Zhao Y., Horowitz J.C., Nho R.S., Mebratu Y.A. (2025). A new era in cancer therapy: Targeting the Proteasome-Bcl-2 axis. J. Exp. Clin. Cancer Res..

[11] Asma S.T., Acaroz U., Imre K., Morar A., Shah S.R.A., Hussain S.Z., Arslan-Acaroz D., Demirbas H., Hajrulai-Musliu Z., Istanbullugil F.R. (2022). Natural Products/Bioactive Compounds as a Source of Anticancer Drugs. Cancers.

[12] Naik R., Harmalkar D.S., Xu X., Jang K., Lee K. (2015). Bioactive benzofuran derivatives: Moracins A-Z in medicinal chemistry. Eur. J. Med. Chem..

[13] Gao C., Sun X., Wu Z., Yuan H., Han H., Huang H., Shu Y., Xu M., Gao R., Li S. (2020). A Novel Benzofuran Derivative Moracin N Induces Autophagy and Apoptosis Through ROS Generation in Lung Cancer. Front. Pharmacol..

[14] Zhong X., Ke X., Yang H., Ye X., Li C., Pan J., Ran W., Wang F., Cui H. (2024). Moracin D suppresses cell growth and induces apoptosis via targeting the XIAP/PARP1 axis in pancreatic cancer. Phytomedicine.

[15] Hwang S.M., Lee H.-J., Jung J.H., Sim D.Y., Hwang J., Park J.E., Shim B.S., Kim S.-H. (2018). Inhibition of Wnt3a/FOXM1/β-Catenin Axis and Activation of GSK3β and Caspases Are Critically Involved in Apoptotic Effect of Moracin D in Breast Cancers. Int. J. Mol. Sci..

[16] Yoon J.S., Lee H.J., Sim D.Y., Im E., Park J.E., Park W.Y., Koo J.I., Shim B.S., Kim S.H. (2021). Moracin D induces apoptosis in prostate cancer cells via activation of PPAR gamma/PKC delta and inhibition of PKC alpha. Phytother. Res..

[17] Dougherty M.W., Hoffmann R.M., Hernandez M.C., Airan Y., Gharaibeh R.Z., Herzon S.B., Yang Y., Jobin C. (2025). Genome-scale CRISPR/Cas9 screening reveals the role of PSMD4 in colibactin-mediated cell cycle arrest. mSphere.

[18] Zhou P., Du X., Jia W., Feng K., Zhang Y. (2024). Engineered extracellular vesicles for targeted reprogramming of cancer-associated fibroblasts to potentiate therapy of pancreatic cancer. Signal Transduct. Target. Ther..

[19] Dehghan-Nayeri M.J., Peytam F., Foroumadi A., Mahdavi M. (2025). Synthesis, characterization, anti-proliferative, and apoptotic activity of a novel quinazoline-containing 1,2,3-triazole toward three human cancer cells. Sci. Rep..

[20] Ye Q., Wu Y., Kuang D., Ye H. (2025). Phytochemical analysis and molecular mechanisms of anticancer activity of *Stevia rebaudiana* extract in gastric cancer cells: In silico docking and functional assays on apoptosis and cell migration. Kuwait J. Sci..

[21] Vogler M., Braun Y., Smith V.M., Westhoff M.-A., Pereira R.S., Pieper N.M., Anders M., Callens M., Vervliet T., Abbas M. (2025). The BCL2 family: From apoptosis mechanisms to new advances in targeted therapy. Signal Transduct. Target. Ther..

[22] Kwon J.W., Oh J.S., Seok S.H., An H.W., Lee Y.J., Lee N.Y., Ha T., Kim H.A., Yoon G.M., Kim S.E. (2023). Combined inhibition of Bcl-2 family members and YAP induces synthetic lethality in metastatic gastric cancer with RASA1 and NF2 deficiency. Mol. Cancer.

[23] Sethy C., Kundu C.N. (2021). 5-Fluorouracil (5-FU) resistance and the new strategy to enhance the sensitivity against cancer: Implication of DNA repair inhibition. Biomed. Pharmacother..

[24] Biagioni A., Peri S., Versienti G., Fiorillo C., Becatti M., Magnelli L., Papucci L. (2023). Gastric Cancer Vascularization and the Contribution of Reactive Oxygen Species. Biomolecules.

[25] Choi Y., Kim Y., Boo H.J., Yoon D., Cha J.S., Yoo J. (2025). Natural Product-Derived Drugs: Structural Insights into Their Biological Mechanisms. Biomolecules.

[26] Socaciu M.A., Diaconeasa Z., Rugina D., Socaciu C., Moldovan R., Clichici S. (2026). Mechanistic Insights into the Metabolic Pathways and Neuroprotective Potential of Pentacyclic Triterpenoids: In-Depth Analysis of Betulin, Betulinic, and Ursolic Acids. Biomolecules.

[27] Ding Y., Yu Y. (2025). Therapeutic potential of flavonoids in gastrointestinal cancer: Focus on signaling pathways and improvement strategies (Review). Mol. Med. Rep..

[28] Su J., Thakur A., Pan G., Yan J., Gaurav I., Thakur S., Yang Z., Cili A., Zhang K. (2023). Morus alba derived Kuwanon-A combined with 5-fluorouracil reduce tumor progression via synergistic activation of GADD153 in gastric cancer. MedComm Oncol..

[29] Azzam H.N., El-Dessouki A.M., Attallah K.A., Sadek M.A., Aboulmagd Y.M., Hassan M.-A.M., Fahmy M.I., El-Shiekh R.A., Kamal R.M., Khalifa H.O. (2025). Morusin as a drug candidate: Opportunities, limitations, and the path toward clinical translation. Front. Pharmacol..

[30] Liu Y., Tang A., Liu M., Luo Z., Cao F., Yang C. (2025). The effectiveness of sanggenon c in alleviating SLC7A11-induced ferroptosis in lung cancer was evaluated using in vivo, in vitro, and computational approaches. Int. Immunopharmacol..

[31] Liu J., Peng Y., Wei W. (2022). Cell cycle on the crossroad of tumorigenesis and cancer therapy. Trends Cell Biol..

[32] Ghafouri-Fard S., Khoshbakht T., Hussen B.M., Dong P., Gassler N., Taheri M., Baniahmad A., Dilmaghani N.A. (2022). A review on the role of cyclin dependent kinases in cancers. Cancer Cell Int..

[33] Ghani M.U., Shi J., Du Y., Zhong L., Cui H. (2024). A comprehensive review on the dynamics of protein kinase CK2 in cancer development and optimizing therapeutic strategies. Int. J. Biol. Macromol..

[34] Yuan X., Li J., Yu B., Cai F., Chen B., Liu J., Peng Y., Zeng D., Liao Q., Liu L. (2025). Demethylzeylasteral inhibits proliferation and metastasis of osteosarcoma cells by modulating the PI3K/AKT/Autophagy pathways. J. Bone Oncol..

[35] Cvetanova B., Li M.-Y., Yang C.-C., Hsiao P.-W., Yang Y.-C., Feng J.-H., Shen Y.-C., Nakagawa-Goto K., Lee K.-H., Shyur L.-F. (2021). Sesquiterpene Lactone Deoxyelephantopin Isolated from *Elephantopus scaber* and Its Derivative DETD-35 Suppress BRAFV600E Mutant Melanoma Lung Metastasis in Mice. Int. J. Mol. Sci..

[36] Wang Q., Bode A.M., Zhang T. (2023). Targeting CDK1 in cancer: Mechanisms and implications. npj Precis. Oncol..

[37] Suski J.M., Braun M., Strmiska V., Sicinski P. (2021). Targeting cell-cycle machinery in cancer. Cancer Cell.

[38] King L.E., Hohorst L., García-Sáez A.J. (2023). Expanding roles of BCL-2 proteins in apoptosis execution and beyond. J. Cell Sci..

[39] Sarosiek K.A., Wood K.C. (2023). Endogenous and imposed determinants of apoptotic vulnerabilities in cancer. Trends Cancer.

[40] Otieno M., Powrózek T., García-Foncillas J., Martinez-Useros J. (2025). The crosstalk within tumor microenvironment and exosomes in pancreatic cancer. Biochim. Biophys. Acta (BBA) Rev. Cancer.

[41] Kaloni D., Diepstraten S.T., Strasser A., Kelly G.L. (2023). BCL-2 protein family: Attractive targets for cancer therapy. Apoptosis.

[42] Iksen, Witayateeraporn W., Hardianti B., Pongrakhananon V. (2024). Comprehensive review of Bcl-2 family proteins in cancer apoptosis: Therapeutic strategies and promising updates of natural bioactive compounds and small molecules. Phytother. Res..

[43] Alam M., Ali S., Mohammad T., Hasan G.M., Yadav D.K., Hassan M.I. (2021). B Cell Lymphoma 2: A Potential Therapeutic Target for Cancer Therapy. Int. J. Mol. Sci..

[44] Zhang N., Yin Y., Xu S.-J., Chen W.-S. (2008). 5-Fluorouracil: Mechanisms of Resistance and Reversal Strategies. Molecules.

[45] Powrózek T., Otieno M., Maffeo D., Frullanti E., Martinez-Useros J. (2025). Blood circulating miRNAs as pancreatic cancer biomarkers: An evidence from pooled analysis and bioinformatics study. Int. J. Biol. Macromol..

[46] Lacalle-Gonzalez C., Lopez-Blazquez C., Hidalgo-Leon M.A., Otieno M.O., Sanz-Criado L., Fernandez-Aceñero M.J., Ortega-Medina L., Garcia-Foncillas J., Martinez-Useros J. (2025). FOLFIRINOX Combined with GPX4 Inhibition Induces Ferroptosis and Defines Redox-Based Therapeutic Subgroups in Pancreatic Cancer. bioRxiv.

[47] Yang Y., Han J., Ma Y., Zhang J., Zhang Z., Wang G. (2020). Demethylzeylasteral inhibits cell proliferation and enhances cell chemosensitivity to 5-fluorouracil in Colorectal Cancer cells. J. Cancer.

[48] Yang H., Huang S., Wei Y., Cao S., Pi C., Feng T., Liang J., Zhao L., Ren G. (2017). Curcumin Enhances the Anticancer Effect of 5-fluorouracil against Gastric Cancer through Down-Regulation of COX-2 and NF- κB Signaling Pathways. J. Cancer.

