# Supplementary material for: Moracin D Inhibits Gastric Cancer Progression Through B-Cell Lymphoma-2 (Bcl-2)-Mediated Cell Cycle Arrest and Apoptosis, Enhancing Chemotherapy Efficacy
Source: Biomolecules. 2026 Mar 13;16(3):428. doi: 10.3390/biom16030428 (PMC13023619; doi:10.3390/biom16030428)
Supplement: Supplementary file 1 [file biomolecules-16-00428-s001.zip › biomolecules-4120864-supplementary.pdf]

Supplementary Material

1. Supplementary Materials and Methods

Table S1. Antibodies information used for Western blot.

**Antibody name**	**Dilution**	**Supplier**	**Cat. No**
Cyclin B1	1:2000	Proteintech (Wuhan, China)	55004-1-AP
CDK1	1:2000	Proteintech (Wuhan, China)	19532-1-AP
α-Tubulin	1:10000	Proteintech (Wuhan, China)	80762-1-RR
PARP	1:1000	Cell Signaling Technology (USA)	9532T
cleaved-PARP	1:1000	Cell Signaling Technology (USA)	5625T
cleaved-caspase-3	1:1000	Cell Signaling Technology (USA)	9664T
cleaved-caspase-9	1:1000	Cell Signaling Technology (USA)	20750
Bax	1:1000	Cell Signaling Technology (USA)	14796T
Bcl-2	1:1000	Abcam	ab32124
HRP- conjugated Goat anti-mouse	1:1000	Beyotime (China)	A0126
HRP- conjugated Goat anti-rabbit	1:1000	Beyotime (China)	A0208

2. Supplementary Results

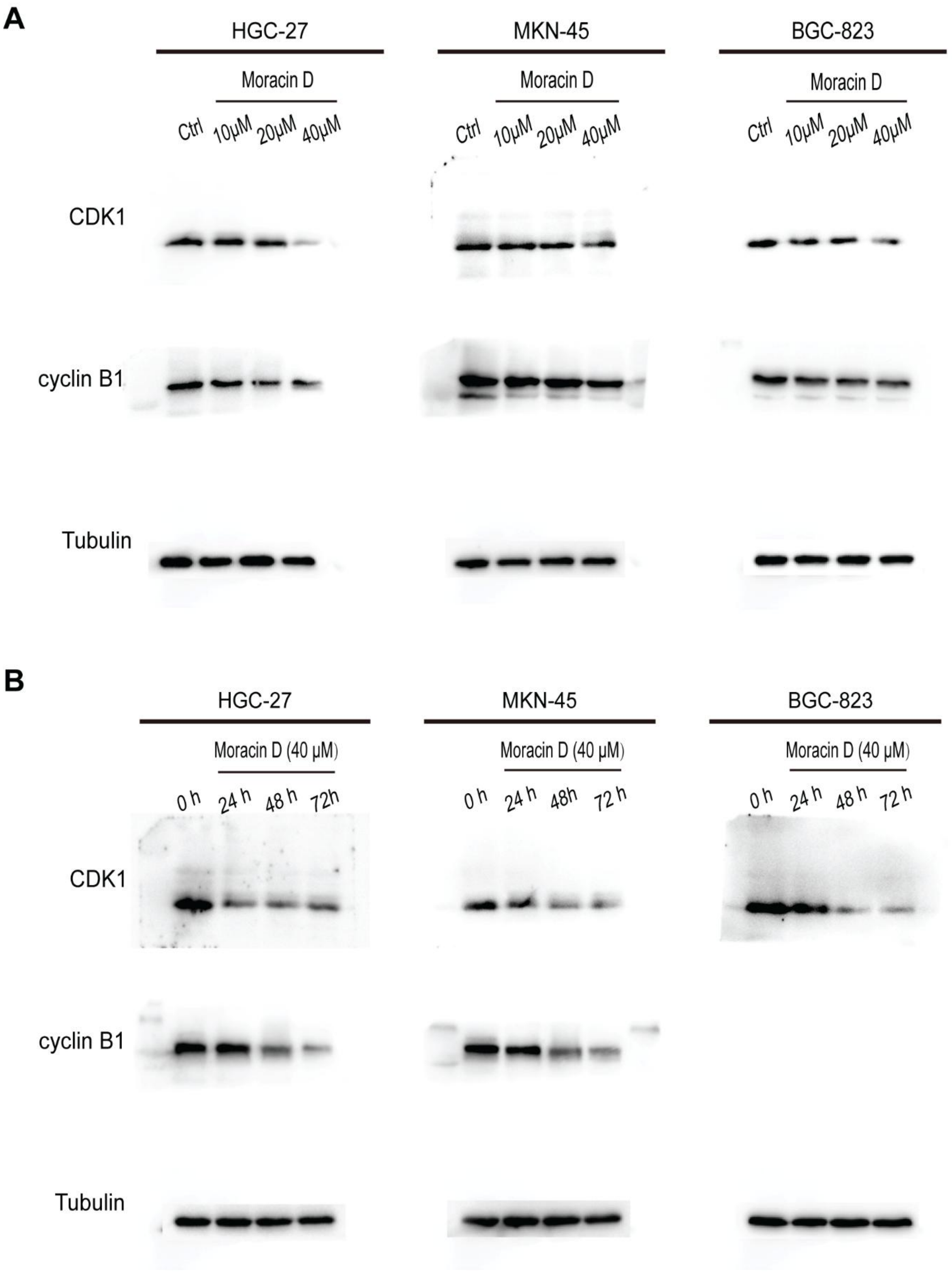

Figure S1. The original WB images of Figures 3 B and D.

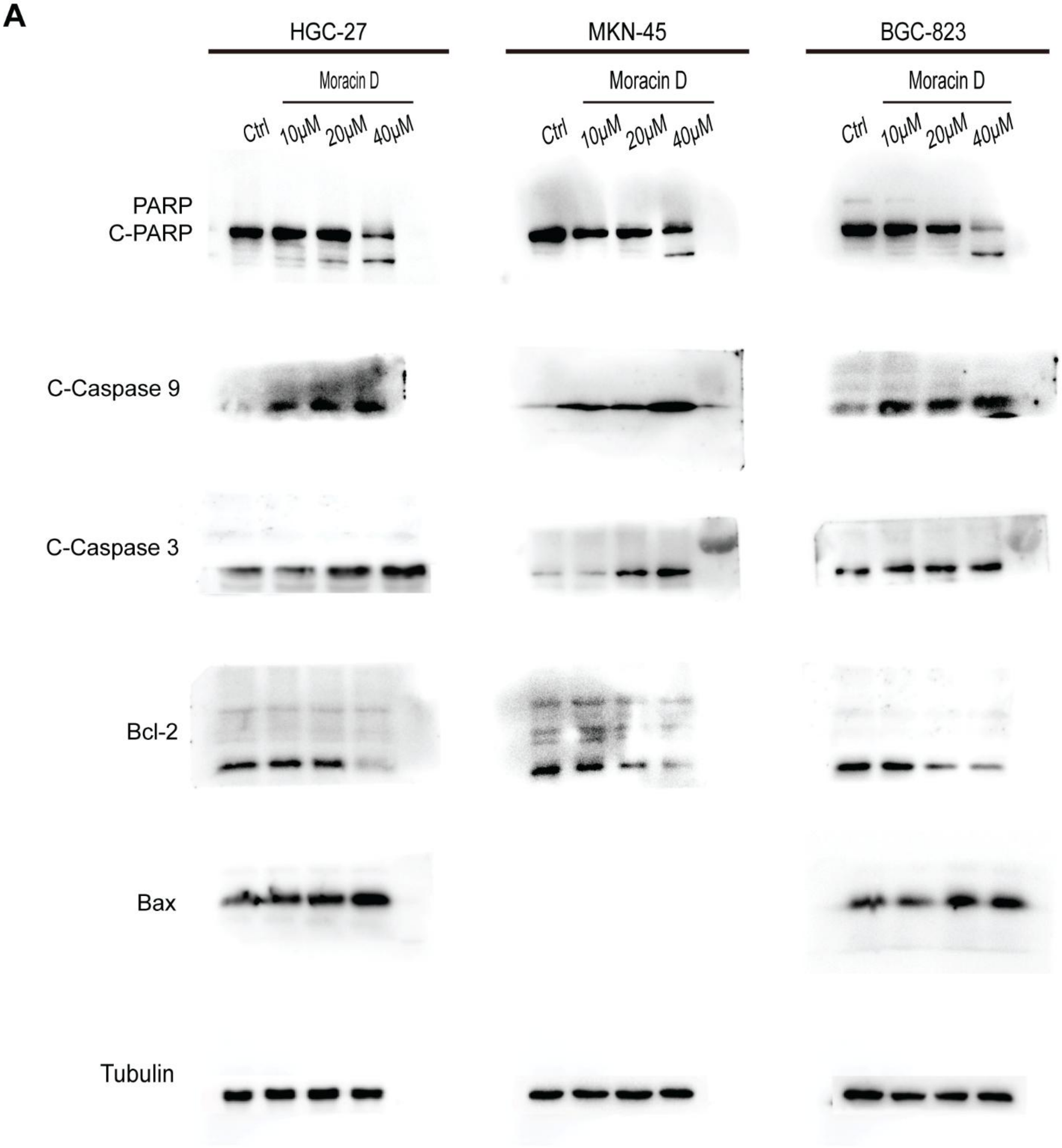

Figure S2. The original WB images of Figure 4 B.

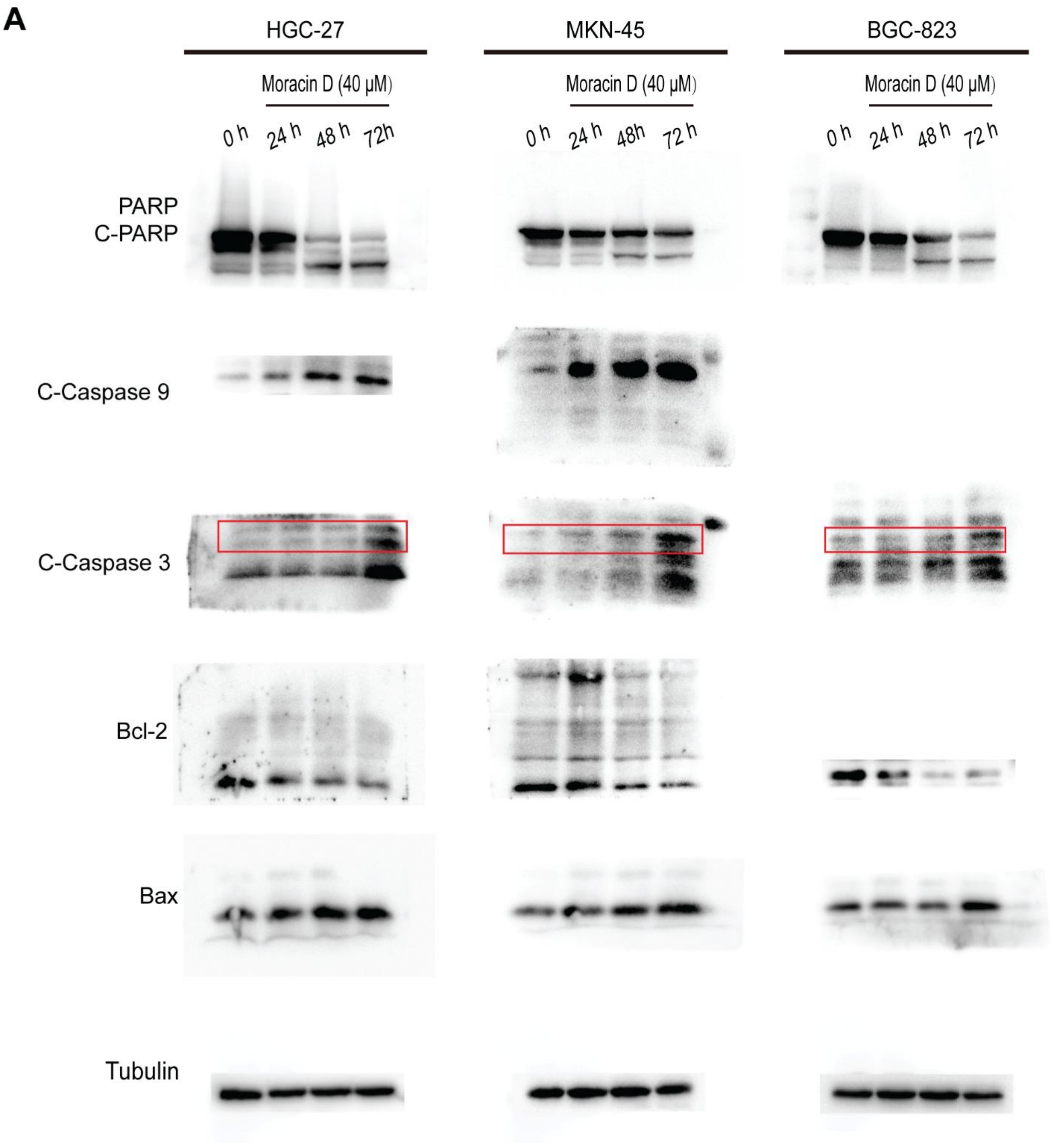

Figure S3. The original WB images of Figure 4 D.

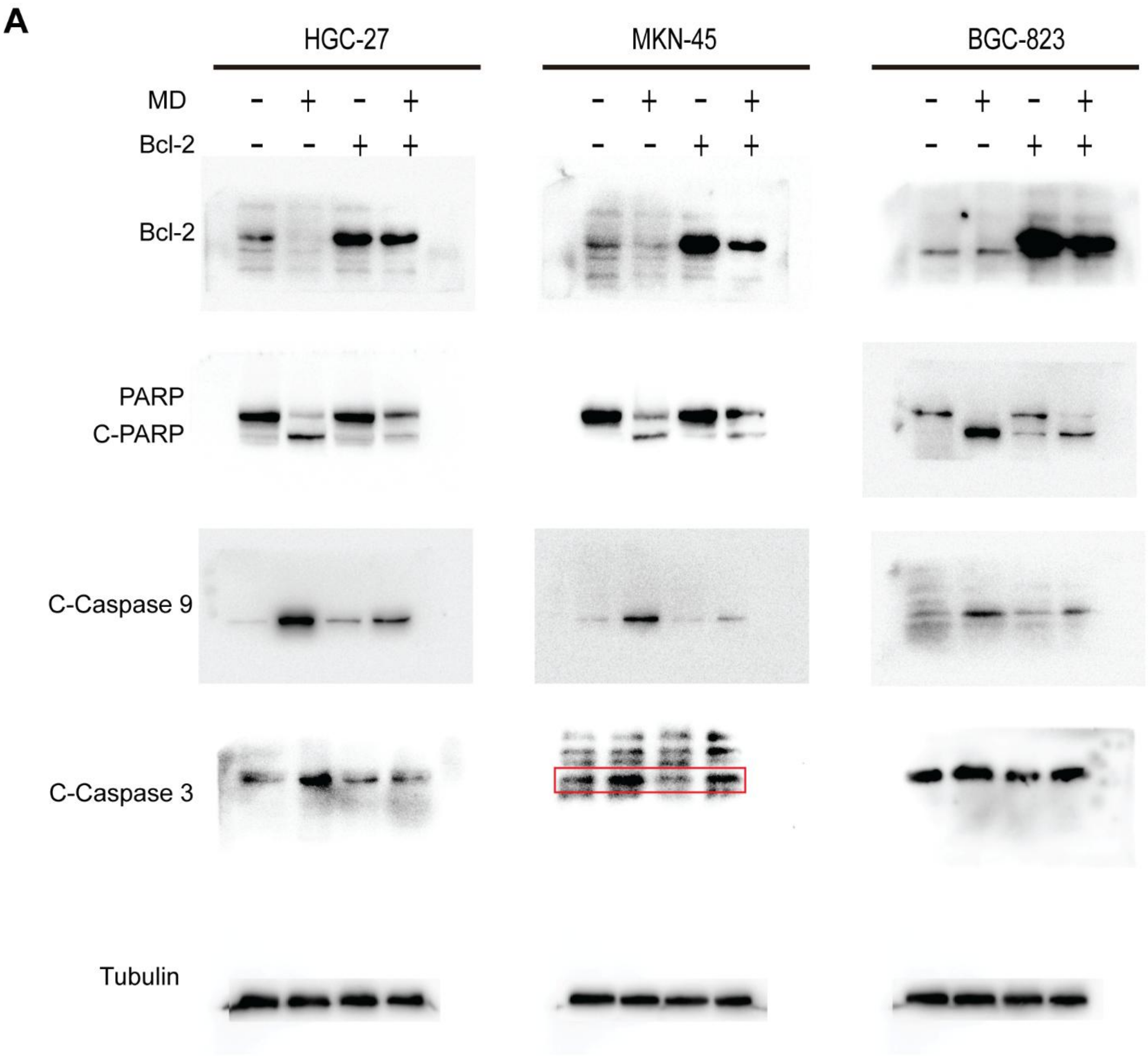

Figure S4. The original WB images of Figure 6 D.

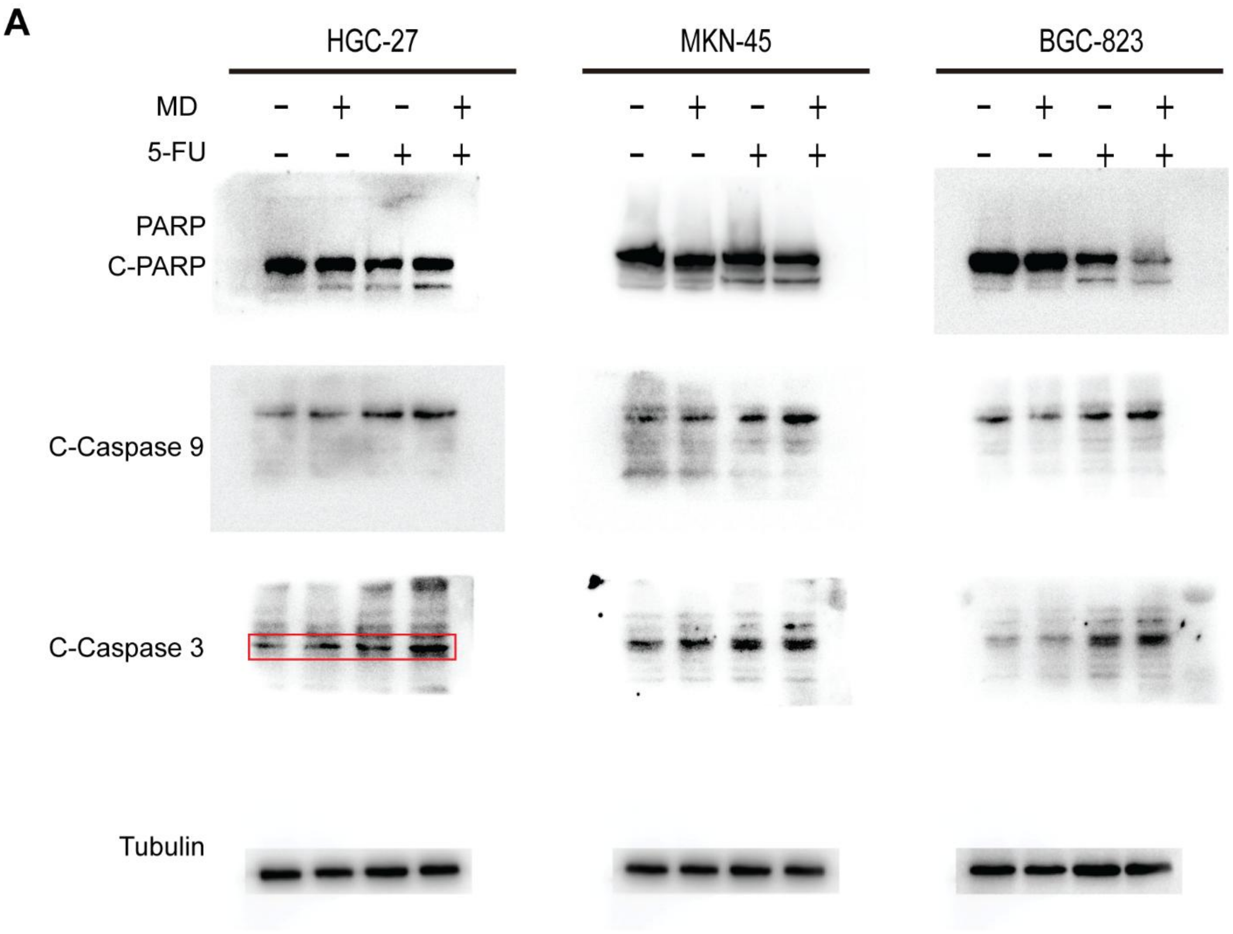

Figure S5. The original WB images of Figure 7 E.

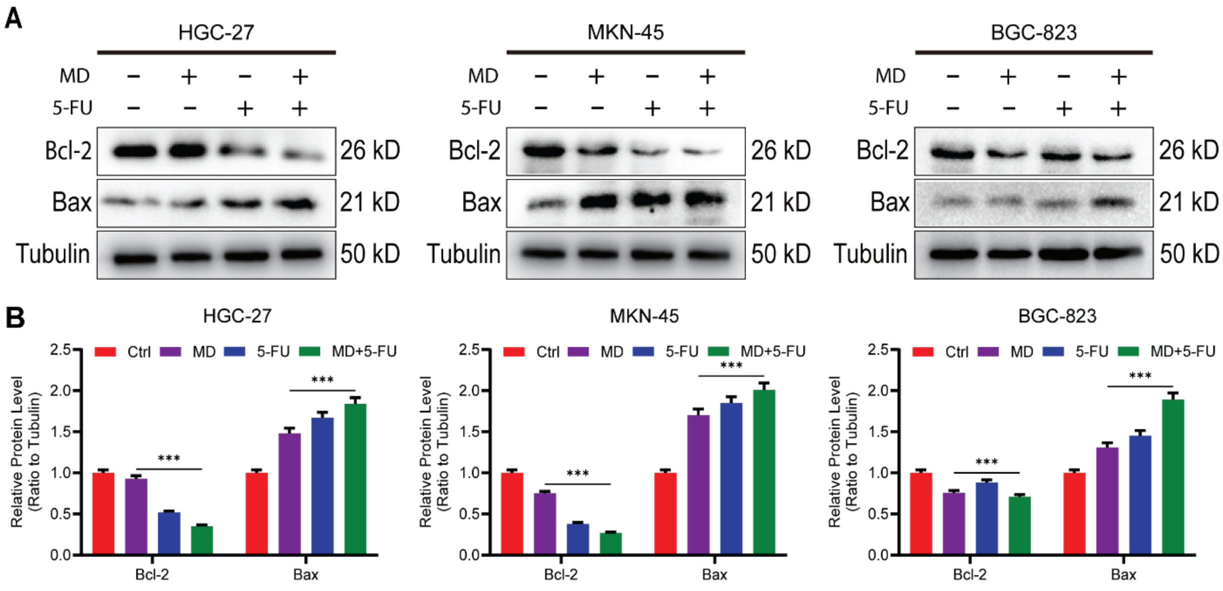

Figure S6. Bcl-2 and Bax protein expression in GC cells. (**A**) WB analysis of Bcl-2 and Bax in GC cells

treated with MD (10 μM), 5-FU (5 μM), or their combination for 48 hours. (**B**) Densitometric quantification of the WB results from panel D.
